# Pulmonary Embolism: A Complication of COVID-19 or Drug Abuse?

**DOI:** 10.7759/cureus.69821

**Published:** 2024-09-20

**Authors:** Manish Gaba, Naveen Kumar, Ankita Pandey, Bijjam Venkata Vijay Kumar Reddy, Arun Dewan

**Affiliations:** 1 Internal Medicine, Max Smart Super Speciality Hospital, New Delhi, IND

**Keywords:** covid-19, deep vein thrombosis (dvt), general internal medicine, marijuana abuse, mental health and covid-19, pulmonary embolism (pe)

## Abstract

Our patient was a young male who had presented to the emergency room with complaints of shortness of breath for three days. His workup was suggestive of a pulmonary embolism. The patient had a history of COVID-19 pneumonia six months prior to this admission, from which he recovered completely. He denied use of recreational marijuana (smoking or edibles initially) but later accepted to smoking marijuana. In fact, he had increased the dose and frequency over the last six months. The COVID-19 infection had a strong impact on his mental health, which pushed him to increase marijuana use. Our case report highlights drug abuse as a risk factor for venous thromboembolism, confounded by the recent COVID-19 infection.

## Introduction

This case study discusses the impact of marijuana abuse on a patient who had recovered from COVID-19 infection. He presented to us with complaints of shortness of breath for three days. He had recovered from COVID-19 pneumonia of moderate severity six months ago. He had adiposity-based chronic disease and no other known comorbidities. The initial impression was that the pulmonary embolism could be associated with long COVID and the prothrombotic state associated with it [1]. However, the patient had completely recovered from his illness. He did not have any thromboembolic complications (TECs) during his COVID-19 infection. He did not have a family history of thromboembolism. He was prescribed anticoagulation post his COVID-19 infection based on elevated D-dimer and an International Medical Prevention Registry on Venous Thromboembolism (IMPROVE)-D score of three. This is in line with the recommendation of the MICHELLE trial regarding post-discharge thromboprophylaxis in COVID-19-related hospitalization [2]. A detailed reevaluation of his history revealed a significant consumption of cannabis in the form of marijuana smoking. We believe this to be the cause of the pulmonary embolism.

Cannabis is one of the most frequently used recreational psychoactive substances globally, with an estimated 192 million users of cannabis in 2018, corresponding to 3.9% of the world population [3]. The Ministry of Social Justice and Empowerment's Magnitude of Substance Use in India 2019 survey found that 2.83% of Indians aged between 10 and 75 years (or 31 million people) were current users of cannabis products [4].

This case report presents an interesting opportunity to learn more about pulmonary embolisms and cannabis use. While several case reports have been cited [5-6], there are no research studies on this topic showing a lacuna in the literature. This is an important risk factor for venous thromboembolism. This becomes more relevant in the background of increasing cannabis use all over the world.

## Case presentation

A male in his 20s with adiposity-based chronic disease and no other comorbidities came to the emergency room with complaints of progressively worsening exertional shortness of breath for three days. At the time of admission, he was breathless at rest. There was no history of orthopnea or paroxysmal nocturnal dyspnea. It was associated with chest tightness and generalized weakness. He did not have any history of fever, cough, sputum, or any occupational exposures. He was admitted six months ago at another hospital for COVID-19 pneumonia of moderate severity. There was no history of any thromboembolic events while he was admitted for COVID-19 pneumonia. He did not have a family history of thromboembolic events. He used to drink alcohol on social occasions, mostly whiskey or vodka, about 300 ml per sitting. Also, he would smoke cigarettes only when he was consuming alcohol, about six to eight sticks each time. This social indulgence usually occurred two to three times per week. On clinical examination, the patient’s heart rate was elevated at 110 beats per minute, blood pressure was 120/80 mmHg, respiratory rate was 25 breaths per minute, and SpO_2_ was 94% on room air. His cardiovascular examination revealed an elevated jugular venous pressure (JVP) and loud second heart sound (S2) on auscultation, and chest examination revealed bilateral equal air entry on both sides. His abdominal and neurological examinations were normal.

Investigation

The patient was admitted to the cardiology intensive care unit. Electrocardiography (ECG) was done in the emergency room, which revealed sinus tachycardia with T-wave inversion in anterior chest leads. The patient's complete blood counts and biochemistry are mentioned in Table 1.

**Table 1:** Laboratory investigations ESR: erythrocyte sedimentation rate; SGOT: serum glutamic oxaloacetic transaminase; SGPT: serum glutamate pyruvate transaminase; ALP: alanine aminotransferase; GGT: gamma-glutamyl transpeptidase; NTproBNP: N-terminal Pro B type natriuretic peptide; CK-MB: creatine kinase-myocardial band

Investigation	Value	Reference range
Hemoglobin (g/dl)	14	13-7
Total leucocyte count (cell/cumm)	5000	4-10
Platelet (cell/cumm)	250000	150-410
Creatinine (mg/dl)	1	0.9-1.3
Sodium (mmol/L)	138	136-146
Potassium (mmol/L)	4.3	3.5-5.1
Calcium (mg/dl)	9	8.8-10.2
SGOT (IU/L)	30	15-41
SGPT (IU/L)	40	17-63
ALP (IU/L)	122	32-91
GGT (IU/L)	65	7-50
Total bilirubin (mg/dl)	0.7	0.3-1.2
Albumin (g/dl)	4	3.5-5.0
Troponin-I (ng/ml)	0.46	0-0.04
CK-MB (ng/ml)	6.7	0.6-6.3
D-dimer(ng/ml)	687	0-243
NT-pro-BNP (pg/ml)	3880	0-450
Protein C (%)-done after 6 weeks on follow-up	120	70-140
Protein S (%)-done after 6 weeks on follow-up	108.7	74-146
Factor V leiden mutation	not detected	
Prothrombin gene mutation	not detected	
Antithrombin III activity (%)	90	80-130

**Figure 1:**
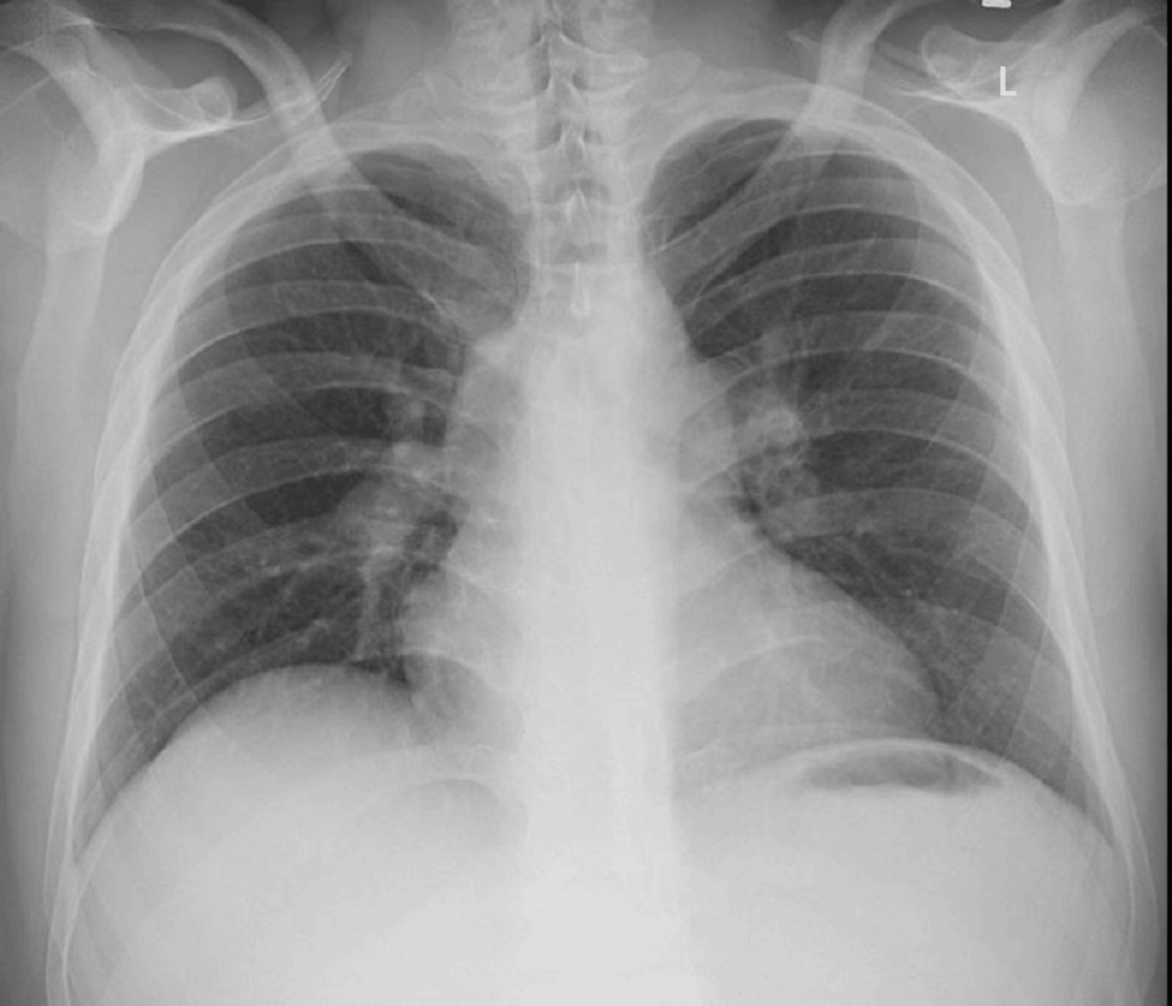
Chest radiograph in posteroanterior view with no significant abnormality

His D-dimer was elevated. The troponin-I, creatine kinase-myocardial band (CK-MB), and NT-pro-BNP were raised. An echocardiography was done, which revealed right atrial and ventricle enlargement with pulmonary artery pressure of 58 mmHg and no regional wall motion abnormality. The left ventricular ejection fraction was 50%-55%. The patient’s chest X-ray in posteroanterior view showed no focal lesion in the lung parenchyma (Figure 1).

**Figure 2:**
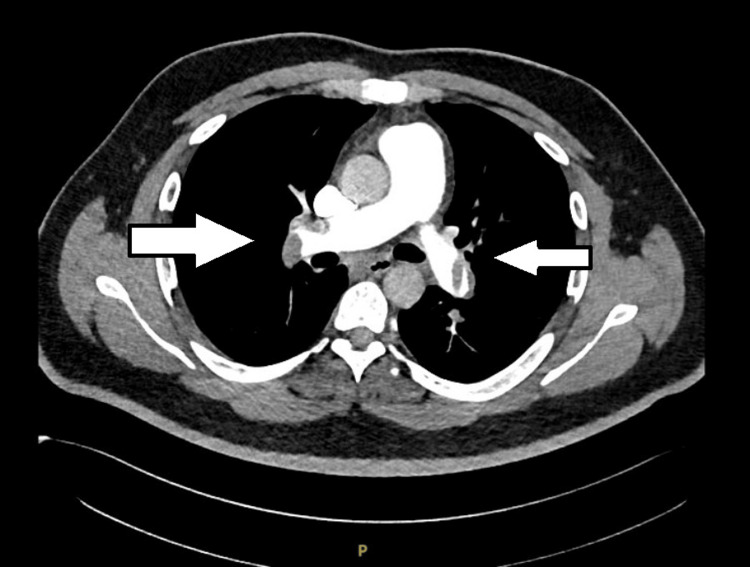
The patient's CT pulmonary angiography (axial view) shows multiple filling defects in superior and inferior segmental branches of both pulmonary arteries, suggestive of acute pulmonary thromboembolism.

**Figure 3:**
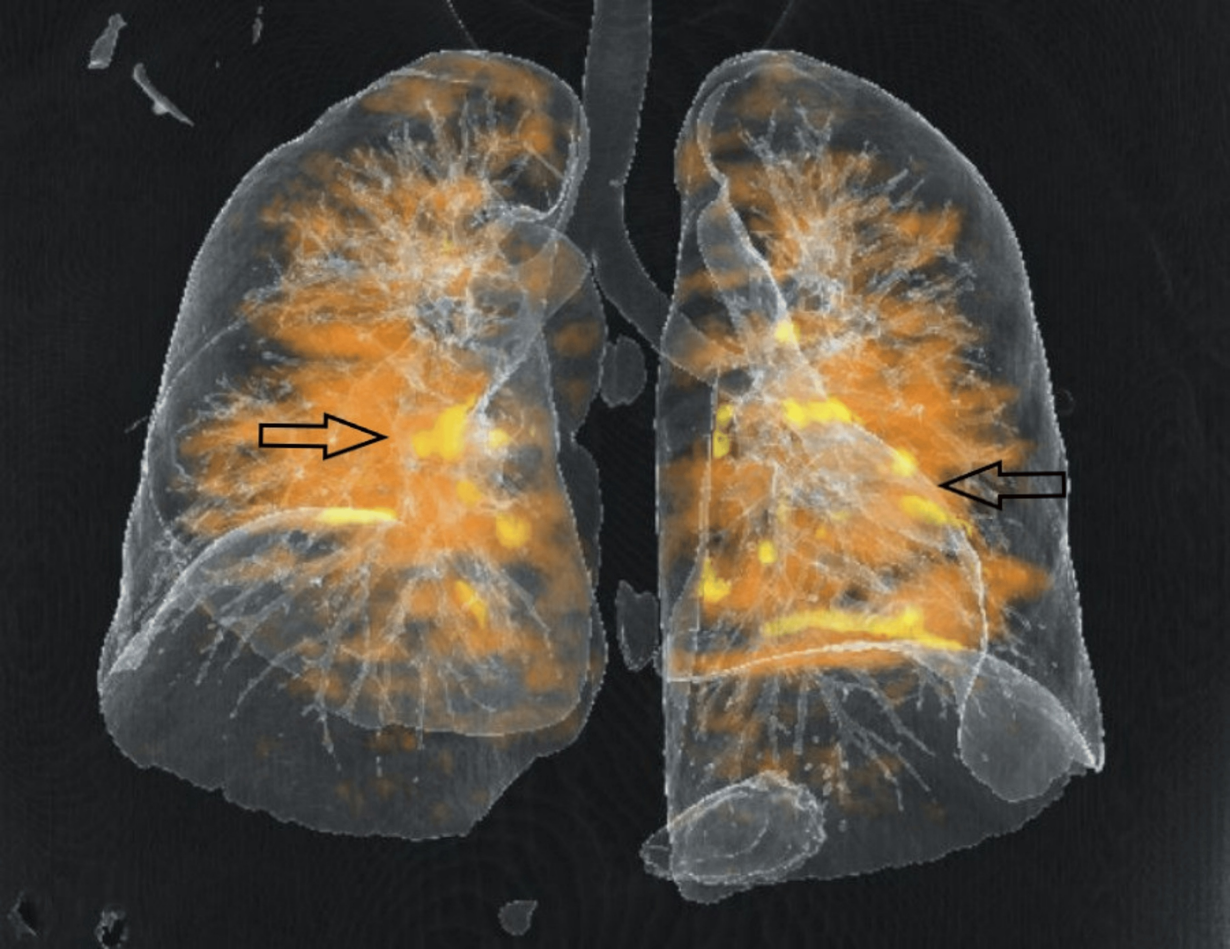
Software-rendered 3D reconstruction of bilateral pulmonary arteries with embolism

The real-time reverse transcription-polymerase chain reaction (RT-PCR) for COVID-19 was negative. The CT pulmonary angiography revealed multiple filling defects in superior and inferior segmental branches of both pulmonary arteries suggestive of pulmonary thromboembolism (Figures 2-3).

His lower limb Doppler revealed no evidence of deep vein thrombosis.

Differential diagnosis

The patient was initially suspected to be a case of acute coronary syndrome. He had elevated levels of D-dimer and troponin-I. However, his echocardiography revealed right atrial and ventricle enlargement with no regional wall motion abnormality. Therefore, suspecting a pulmonary embolism, a CT pulmonary angiography was done, which confirmed the diagnosis. He had undergone a chest CT during his previous admission. The records of this showed findings suggestive of COVID-19 pneumonia and no evidence of pulmonary embolism. This chest CT was done at another hospital, and the films of the same were not available to us. As for etiology, initially, we thought it to be associated with long COVID [1]. It is well established that COVID-19 is a pro-coagulant and pro-inflammatory state that can precipitate pulmonary embolism [7]. Our patient had a COVID-19 infection six months ago, following which he had taken anticoagulants for a period of six weeks. Apixaban was prescribed at a dose of 2.5 mg twice daily in view of prolonged hospital stay, COVID-19 infection of moderate severity, and elevated D-dimer values.

The patient’s International Medical Prevention Registry on Venous Thromboembolism (IMPROVE)-D score for the venous thromboembolism score was three [8]. The findings of the MICHELLE trial recommend post-discharge thromboprophylaxis. He had no family history of thromboembolic events or pro-coagulant disorders [2]. At this point, the patient’s history was revisited, and he was asked about recreational drug use, if any. He gave a history of smoking weed (marijuana) and hash (concentrated marijuana). For the previous six months, he was regularly consuming weed, hash, cigarettes, and alcohol since recovering from the COVID-19 infection. Post-COVID, he was constantly worried about the uncertainty of life in the background of the pandemic and the long-term health impact of his COVID-19 infection. He would relieve his stress by indulging in these activities. The patient was evaluated after six weeks for protein C and protein S deficiency, factor V Leiden mutation, and antithrombin III deficiency, which were negative. This led us to attribute the pulmonary embolism to cannabis use in the form of smoking.

Treatment

The patient was shifted to the critical care unit for further management. In the ICU, he was found to be hypotensive with a blood pressure of 96/60 mmHg, a significant fall from his baseline, and his SpO_2_ was 88% on room air. Because of hemodynamic compromise and respiratory distress, the patient was taken up for thrombolysis with ateleptase. Post thrombolysis, his symptoms improved, right side chamber enlargement reduced, and pulmonary artery pressure improved to 35 mmHg. He was subsequently started on anticoagulation with apixaban at a dose of 5 mg twice daily. On discharge, he was advised to follow up with a psychiatrist to help with de-addiction and manage his anxiety.

Follow-up

The patient was symptomatically better in the follow-up. He had stopped smoking cannabis. He was kept on anticoagulation with apixaban for three months. A repeat CT pulmonary angiogram done after one year at the patient’s insistence revealed a resolution of pulmonary embolism.

## Discussion

This is an interesting case of pulmonary embolism. The patient did not have any risk factors for venous thromboembolism. The initial thought of long COVID was considered but unlikely in view of his complete symptomatic recovery. The history of cannabis consumption in the form of smoking was significant. It represents an important risk factor for venous thromboembolism, especially in the background of increased cannabis consumption all over the world.

Our patient had a history of occasionally indulging in these harmful habits. However, to deal with the stress and anxiety associated with a COVID-19 infection, he increased his cannabis use significantly, thus resulting in this complication. The psychological impact of COVID-19 has been an increase in drug use, particularly cannabis. Its online sales increased in the first three months of 2020 during the start of the pandemic [9]. A large proportion of those who used cannabis in the past had increased their consumption during the pandemic [10]. There is a need for interventions and educational campaigns to limit the use of cannabis. The National Action Plan for Drug Demand Reduction (NAPDDR) is a centrally sponsored scheme being implemented by the Ministry of Social Justice & Empowerment that aims to address this problem in India by providing financial assistance for de-addiction centers, educational campaigns, and vocational training for ex-drug addicts.

Marijuana has a pro-coagulant effect. This is attributed to the presence of CB1R and CB2R on human platelets. There is an increase in the surface expression of glycoprotein IIb-IIIa and P-selectin in individuals with marijuana use [11]. Activated CB1R causes endothelial dysfunction, which leads to the development of atherosclerosis. CB1R stimulation in endothelial cells activates the MAPK pathway, which triggers the release of reactive oxygen species (ROS) [12].

These effects lead to endothelial dysfunction and a pro-coagulant state. Highlighting the pro-coagulant effect of marijuana, Farouji I et al. reported an interesting case of a female in her 40s who presented with ascending aortic thrombus, associated with acute arterial occlusion of the right vertebral artery and bilateral renal artery. The only risk factor this patient had was marijuana smoking [5]. Another case was reported by Eikowski W et al., where a male in his 20s presented with severe pulmonary embolism without having any cardiovascular risk factors [6]. These case reports reveal a pattern of increased embolic phenomenon with marijuana use, especially in younger individuals.

A study done by Stupinski et al. revealed an increased risk of TECs in patients of trauma with a history of tetrahydrocannabinol (THC) use as compared with those not using THC (3.5% vs. 1.1%, p = 0.03). The rates of deep venous thrombosis (6.6% vs. 1.8%, p = 0.02) and pulmonary embolism (2.2% vs. 0.2%, p = 0.04) were higher in the group using THC [13]. Another study done on trauma patients in the geriatric age group revealed similar findings [14]. Users of THC had significantly higher rates of TEC compared to THC non-users (3.0% vs. 1.7%; p = 0.01). Rates of deep vein thrombosis (2.2% vs. 0.6%, p < 0.01) and pulmonary embolism (1.4% vs. 0.4%, p < 0.01) were higher in the THC users [13]. In another Mendelian randomized trial by Chen et al., increased marijuana use was associated with an increased risk of poor cardiovascular outcomes [15]. The genetic liability to cannabis use disorder was associated with a higher risk of atrial fibrillation, heart failure, pulmonary embolism, and stroke.

Coronavirus disease 2019 is a risk factor for increased venous thromboembolic events. Long COVID is a known entity that can cause a prolonged prothrombotic state. In our patients, the likelihood of long COVID-associated thromboembolic events seems less likely, and cannabis abuse seems to be a more important factor. The management of thromboembolic events and prophylaxis is well known, but the management of the psychological impact of COVID-19 and drug abuse is a topic of ongoing development. We recommend all young adults who have recovered from COVID-19 should be counseled about the harms of drug use, especially marijuana, which is considered by many to be a harmless recreational drug. This becomes more important in individuals who have a moderate or severe COVID-19 infection.

## Conclusions

The COVID-19 pandemic has impacted the health of people all over the world in many ways. While its physiological effects have been extensively studied, the psychological impact has remained neglected. Our case highlights cannabis use as a risk factor for thromboembolism. An increased use of drugs in the post-lockdown phase has become an important public health issue. This is especially important in a young patient presenting with venous thromboembolic complications, and a detailed drug history can be crucial in clinching the diagnosis. Both pulmonary embolism and drug abuse are preventable problems but require significant resources to tackle the problem. We would recommend further studies be conducted to firmly establish the link between cannabis use and venous thromboembolic events.
